# “This Is What We Don't Know”: Treating Epistemic Uncertainty in Bayesian Networks for Risk Assessment

**DOI:** 10.1002/ieam.4367

**Published:** 2020-12-03

**Authors:** Ullrika Sahlin, Inari Helle, Dmytro Perepolkin

**Affiliations:** ^1^ Centre for Environmental and Climate Research Lund University Sweden; ^2^ Faculty of Biological and Environmental Sciences and Helsinki Institute of Sustainability Science (HELSUS) University of Helsinki, Finland, and Natural Resources Institute Finland (Luke) Helsinki

**Keywords:** Epistemic uncertainty, Bayesian network, Uncertainty analysis, Model uncertainty, Subjective probability

## Abstract

Failing to communicate current knowledge limitations, that is, epistemic uncertainty, in environmental risk assessment (ERA) may have severe consequences for decision making. Bayesian networks (BNs) have gained popularity in ERA, primarily because they can combine variables from different models and integrate data and expert judgment. This paper highlights potential gaps in the treatment of uncertainty when using BNs for ERA and proposes a consistent framework (and a set of methods) for treating epistemic uncertainty to help close these gaps. The proposed framework describes the treatment of epistemic uncertainty about the model structure, parameters, expert judgment, data, management scenarios, and the assessment's output. We identify issues related to the differentiation between aleatory and epistemic uncertainty and the importance of communicating both uncertainties associated with the assessment predictions (direct uncertainty) and the strength of knowledge supporting the assessment (indirect uncertainty). Probabilities, intervals, or scenarios are expressions of direct epistemic uncertainty. The type of BN determines the treatment of parameter uncertainty: epistemic, aleatory, or predictive. Epistemic BNs are useful for probabilistic reasoning about states of the world in light of evidence. Aleatory BNs are the most relevant for ERA, but they are not sufficient to treat epistemic uncertainty alone because they do not explicitly express parameter uncertainty. For uncertainty analysis, we recommend embedding an aleatory BN into a model for parameter uncertainty. Bayesian networks do not contain information about uncertainty in the model structure, which requires several models. Statistical models (e.g., hierarchical modeling outside the BNs) are required to consider uncertainties and variability associated with data. We highlight the importance of being open about things one does not know and carefully choosing a method to precisely communicate both direct and indirect uncertainty in ERA. *Integr Environ Assess Manag* 2021;17:221–232. © 2020 The Authors. *Integrated Environmental Assessment and Management* published by Wiley Periodicals LLC on behalf of Society of Environmental Toxicology & Chemistry (SETAC)

## KEY POINTS

- We propose a framework for treating epistemic uncertainty that can guide assessors in communicating uncertainty due to limitations in knowledge when using Bayesian networks (BNs) for risk assessment.
- A BN is by itself not enough to characterize uncertainty in an assessment, and uncertainty associated with model structure, expert judgments, data, and management scenarios may require modeling external to a BN.
- There are several ways to characterize direct and indirect epistemic uncertainty, such as a subjective probability, an interval, an uncertainty scenario, or a list of caveats, to be combined with a BN.
- The users of BNs for environmental risk assessment (ERA) should distinguish between aleatory and epistemic BNs and apply expressions and methods for treating uncertainty appropriate for the given type of BN and knowledge bases of the assessment.

## INTRODUCTION

Environmental risk assessment (ERA) is a systematic process of evaluating the impact of natural or anthropogenic threats to organisms. Environmental risk assessment aims to assess risk defined as the probability of adverse events (Burgman 2005; Ayre and Landis 2012; Suter 2016). Knowledge is always limited, and it is impossible to know the probability and severity of an event with absolute certainty (Hansson 2009). Epistemic (knowledge‐based) uncertainty is personal, given that different scientists have different knowledge bases. It is also temporal, given that epistemic uncertainty may change as new information becomes available (Lindley 2006).

Probabilistic risk assessments use probabilistic models to describe the inherent randomness of the physical world, also known as “variability” or “aleatory uncertainty” (Kelly and Smith 2009). Quantitative uncertainty analysis can use probabilities to describe epistemic uncertainty about the assessment model (structure and parameters within the model) (Burgman 2005; EFSA et al. 2018b). The probabilities used for aleatory and epistemic uncertainty represent relative frequencies and subjective probabilities (degree of beliefs), respectively (Apostolakis 1990).

Differentiating between aleatory and epistemic uncertainty in risk assessments is important because it can lead to different results when propagating uncertainty in a model (e.g., Nauta 2000), and more importantly, it allows the uncertainty about an estimated risk to be characterized. Without separation, the probability of an adverse event is a composite probability (Hansson 2008), which can be seen as a mixture of several frequency distributions (corresponding to different parameters or scenarios), weighted by subjective probability. A composite probability is, therefore, an unknown mixture of aleatory and epistemic uncertainty. Quantifying the probability for the adverse event as a composite probability masks an important difference between

- a situation where we are certain about the probability of the adverse event but where the probability is derived from a model with high variability, and
- a situation where we are uncertain whether variability is low or high and therefore uncertain about the probability of the adverse event.

Instead, the methods to treat uncertainty should reflect the knowledge bases used for an assessment (Aven 2010), which includes expressing uncertainty about a conclusion.

In a recent review of practices to communicate epistemic uncertainty in different fields, van der Bles et al. (2019) studied the different practices used to communicate uncertainty about facts, numbers, and scientific hypotheses supporting the assessment (direct epistemic uncertainty), and the strength of the knowledge about them (indirect epistemic uncertainty). Organizations that develop guidance for evidence‐based decision making attempt to improve the way indirect uncertainty is communicated (Morgan et al. 2016). Principles for quantitative risk assessment have, for a long time, primarily focused on direct uncertainty (Apostolakis 1990; Paté‐Cornell 1996). However, both levels of uncertainty are important when making assessments based on quantitative models of complex systems (Paté‐Cornell 2012) and communicating the assessments' conclusions (Morgan and Mellon 2011; Spiegelhalter and Riesch 2011; Institute of Medicine 2013).

Communicating uncertainty may safeguard risk managers from putting too much or too little confidence in the assessment (Hansson 2009; Fischhoff and Davis 2014). Therefore, identifying, characterizing, and communicating uncertainty is an essential part of any risk assessment (FAO WHO 1995; NRC 2009; Mastrandrea et al. 2011; EFSA et al. 2018a, 2019). To communicate about uncertainty effectively, ERA requires methods that can support the communication of uncertainty about the assessed probability of adverse events (direct uncertainty) and the level of confidence in the assessment (indirect uncertainty).

Bayesian networks (BNs) have been used in ERA since the 1990s (Varis 1995; Varis and Kuikka 1997) and have become more popular in the field during the 21^st^ century (Kaikkonen et al. 2020). A BN is a probabilistic model consisting of (chance) nodes (which can represent events or variables of an assessment) and arrows, which typically indicate the direction of causality. The strengths of the relationships between the nodes are quantified using conditional probability distributions, which, taken together, enable the BN to express the joint probability distribution over the nodes.

A BN is a specific type of Bayesian model (Koller and Friedman 2009). A Bayesian model is a joint probability distribution over a network of variables and parameters. This network is based on a directed acyclic graph (DAG). The model's joint probability distribution is based on parametric probability distributions for the marginal or conditional probability distributions of variables and probability distributions for the parameters within them (Kelly and Smith 2009; McElreath 2020). In a BN, variables have a finite number of states (e.g., categorical or discretized continuous), and parameters are not explicit nodes themselves (Koller and Friedman 2009). The strength of BNs lies in the efficient algorithms they use to update the network given an instantiation of nodes (Lauritzen and Spiegelhalter 1988), even when the networks represent large and complex systems.

Bayesian networks have gained popularity because they can model causality (Pearl 2009), which helps achieve meaningful, practical, and coherent analysis (Cox 2013; Fenton and Neil 2018). There are many relatively easy‐to‐use software applications, which make the modeling accessible and ready to use (Scutari and Denis 2014). Many BN software packages represent the assessment model graphically, simplifying communication with nonexpert end users and other stakeholders (Smid et al. 2010; Chen and Pollino 2012).

The present paper aims to clarify how BNs can represent direct epistemic uncertainty and suggests ways to treat uncertainty when using BNs for ERA. Although BNs are probabilistic models, they do not necessarily quantify the relevant epistemic uncertainty, lacking a built‐in ability to differentiate between different types and sources of uncertainty (Chen and Pollino 2012). In ERA and risk assessment in general, there can also be uncertainties not suitable to be characterized by probability.

We conceptualize treatments of epistemic uncertainty when using BNs in risk assessment by distinguishing between 1) the object, source, and level of uncertainty; 2) how uncertainty is expressed; 3) the different types of BNs; and 4) where uncertainties are found in an application of a BN. Then, we apply this framework and identify treatments of uncertainty done within or outside a BN used for risk assessment.

## A FRAMEWORK FOR EPISTEMIC UNCERTAINTY

### What should we be uncertain about?

#### Object

We can be uncertain about “statements of the world, numbers, and scientific models” (van der Bles et al. 2019). “Statements of the world” are either true or false, which can be modeled using categorical variables. “Scientific models” are theories or a mechanistic understanding of how the world works, expressed as mathematical equations representing structural relationships between the variables in the system, system dynamics, and variability. “Numbers” are continuous quantities that can be variables (possible to observe, at least in theory) or parameters. “Parameters” are theoretical constructs defined within a model (cannot be observed directly).

#### Source

There are several sources of uncertainty in risk assessments, such as variability within a sampled population or repeated measurements, computational or systematic inadequacies of measurement, limited knowledge and ignorance about underlying processes, and expert disagreement (van der Bles et al. 2019). It is useful to consider sources associated with 1) data (measurements, observations), 2) models (structural uncertainty and parameter uncertainty), and 3) expert judgments (Regan et al. 2003; Spiegelhalter and Best 2003).

#### Level

Van der Bles et al. (2019) categorize epistemic uncertainty into 2 fundamental levels: “direct” (range of outcomes, likelihoods) and “indirect” (quality of the underlying knowledge, confidence). This distinction is useful because expressions for direct and indirect uncertainty are different. Understanding the indirect uncertainty about the knowledge bases can help the scientists conducting the assessment determine how to use the information and decide on the treatment of direct uncertainty.

### How should we express epistemic uncertainty?

#### Direct epistemic uncertainty

Direct uncertainty can be expressed quantitatively, using numbers or verbal statements, or qualitatively, as indications of directions and magnitudes. In practice, direct uncertainty can be communicated with varying levels of precision, from no communication to certainty expressed by subjective probability (degree of belief) (van der Bles et al. 2019). Quantitative expressions force the assessor to be precise about their uncertainty, which adds transparency to the assessment (EFSA et al. 2018b). A probability distribution can be summarized into less precise expressions such as an expectation, percentile, or probability interval. For example, a quantitative expression of direct epistemic uncertainty about the risk of extinction in a population viability analysis (which is the probability of an adverse event) could be, “we are 90% certain that the risk of extinction is less than 5%.” The meaning of this verbal statement is P(“extinction risk” <5%) = 90% or, when the risk of extinction is expressed as a probability, P(“P_f_(adverse event)” <5%) = 90%. Note that the extinction risk is a summary from an assessment model of aleatory uncertainty, and therefore, we denote the probability for the adverse event by P_f_. A less precise verbal statement would be “we are certain the risk of extinction is less than 5%.” A mutual interpretation of “certain” in terms of subjective probability can be obtained by agreeing on scales that match the probability with verbal terms (Mastrandrea et al. 2011; Spiegelhalter and Riesch 2011; EFSA et al. 2019).

Alternative expressions of direct uncertainty used in ERA are, for example, an interval or a bounded probability (Tucker and Ferson 2003; Burgman 2005). A plain interval is a lower and upper bound on a parameter defining a plausible range. There are several ways to propagate intervals or bounded probabilities on parameters through a model, for example, by searching for bounds on the output under the sets of intervals or using interval analysis or probability bounds analysis.

Another way to express epistemic uncertainty relevant for BN applications in ERA is via uncertainty scenarios. Uncertainty scenarios are used to model events for which we do not have an exhaustive list of possible outcomes or where we do not want to assign a probability to individual outcomes (“don't know” or “can't know” probabilities) (Institute of Medicine 2013). In this case, epistemic uncertainty is not quantified but treated as an “assumption” or “nonmodeled component.”

Assessors derive the answer to the risk assessment questions separately for every scenario, without integration. Each uncertainty scenario is represented by its own BN. In an assessment, uncertainty can be treated by a combination of uncertainty scenarios, for example, alternative model structures, and subjective probabilities expressing epistemic uncertainty in, for example, the parameters, within each uncertainty scenario.

#### Indirect epistemic uncertainty

Assessors often communicate indirect uncertainty as a list of caveats about the underlying sources of evidence, for example, caveats about observations, assumptions, and knowledge elicited from experts (van der Bles et al. 2019). A critical appraisal aims to assess the methodological quality of a study that may influence the reliability of the evidence the study produces (EFSA 2015). In the context of risk assessment, quality appraisal is also performed on the scientific models and assumptions behind the assessment (van der Sluijs et al. 2005). The list of caveats can include a list of criteria with scores similar to the GRADE system (Morgan et al. 2016), which rates and summarizes the risk of different types of biases (Guyatt et al. 2008) or a score for the methods and theory (Gormley et al. 2011, p 35).

Instead of communicating direct and indirect uncertainty side by side, the assessment can allow indirect uncertainty to influence the specification of direct uncertainty. For example, a bound on a subjective probability may express uncertainty about it, indicating the strength of knowledge supporting the quantification of direct epistemic uncertainty (Spiegelhalter and Riesch 2011; Institute of Medicine 2013).

## HOW DO BAYESIAN NETWORKS QUANTIFY UNCERTAINTY?

### A brief story about BNs

Pearl (1988) introduced expert‐informed BNs for probabilistic and causal reasoning. A BN is “Bayesian” in the sense that Bayes' rule is applied for probabilistic reasoning when conditioning the joint probability distribution (i.e., the distribution over all nodes in the network) over a combination of evidence on one or more nodes (an “instantiation,” a “case,” or a “finding” of the network). Evidence, in the context of BNs, is a categorical probability distribution added to a BN node to override the original node state probabilities. Evidence is, in this way, commonly referred to as “virtual evidence,” unless one of the categories is assigned a probability of 1; then, it is called the “hard evidence” (Mrad et al. 2015).

In statistics and computer science, a BN is a model to calculate a likelihood (i.e., the probability of the data given the model and the choice of the values of the parameters within the model) for a network of observable variables. The likelihood is used to select a network structure or a set of parameters with the maximum likelihood (if using frequentist parametric inference) or highest posterior probability (if using Bayesian parametric inference). So‐called “case learning” is the process of selecting parameters of a BN for a given structure (Neapolitan 2004). In this context, BNs are data‐driven models of observable variables where all information about the node state probabilities are included in the probability tables (PTs, nodes without parents) or conditional probability tables (CPTs, nodes with parents). These BNs lack components to express uncertainty in table probabilities (Kontkanen et al. 1998). Therefore, Bayesian parametric inference of a BN is possible only by expanding the table probabilities with a probabilistic model for uncertainty about these values (Koller and Friedman 2009).

Adopting BNs for decision analysis and risk assessment is motivated by the opportunity they provide for modeling a network of variables with a known causal structure and using expert knowledge and/or data to inform the model. Bayesian networks are successful as models for probabilistic reasoning and decision analysis in situations when probabilities inside the PTs and CPTs (table probabilities) are known with certainty. In ERA applications, the probabilities may be known or unknown. Therefore, it is essential to clarify which type of uncertainty the BN is quantifying to understand how to treat and communicate uncertainty.

### Bayesian networks in risk assessment

**Figure 1:**
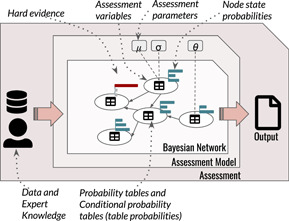
A conceptual model of a Bayesian network used for risk assessment as part of the assessment.

An assessment uses a model informed by data and expert knowledge to produce an output that answers the assessment question (Figure 1). A BN can represent the assessment model, but in ERA, a BN is usually a structure used to link independently developed continuous process‐based models (Borsuk et al. 2004; Carriger and Barron 2020) or a mixture of the two (Landis et al. 2020). The type and the interpretation assigned to the network nodes' probability distributions determine how a BN treats epistemic uncertainty when applied in a risk assessment. Based on the theory and practice of BNs applied in risk assessment, we identify 3 types of BNs:

1) Epistemic BN: Node state probabilities (other than nodes for data) are subjective probabilities that express epistemic uncertainty
2) Aleatory BN: Node state probabilities are relative frequencies that express aleatory uncertainty (variability)
3) Predictive BN: Node state probabilities are a “predictive distribution,” which combines aleatory and epistemic uncertainty.

Combining these 3 types of BNs in the same assessment may be very confusing, especially from the uncertainty communication point of view. We suggest that the type of uncertainty the probability distribution for the nodes (and thereby also the table probabilities) in a BN represents, that is, aleatory or epistemic uncertainty or both, is determined by whether

**Figure 2:**
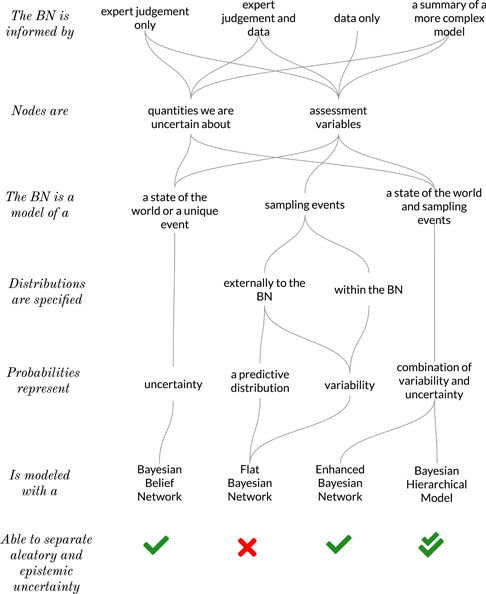
A scheme based on 6 criteria to classify a BN from the point of view of uncertainty. BN = Bayesian network.

- a BN is a model of a unique event or a sampling event,
- the nodes are quantities we are certain or uncertain about, and
- the model was informed by expert judgment, data, or both (Figure 2).

#### Epistemic BN

Epistemic BNs are expert‐informed networks used for probabilistic reasoning in light of the evidence brought into the network. An epistemic BN is a model for a subjective degree of belief where the primary purpose is to make inferences about unknown quantities; therefore, an epistemic BN is a genuine Bayesian belief network (Figure 2).

Nodes in an epistemic BN consist of quantities we are uncertain about, which can be expressed as a true/false statement (e.g., whether a specific person is exposed to a contaminant or not), a number (e.g., the average environmental concentration of a contaminant at a given place during a specified period), or a unique event (Helland 2018). To allow for inference from evidence, an epistemic BN can also have nodes for data or evidence that is conditionally dependent on the quantities we are uncertain about. The table probabilities for nodes in an epistemic BN can be quantified by expert judgment or statistical inference performed outside the BN. The table probabilities for data nodes can also be learned inside the BN.

Epistemic BNs have been used to conclude the current status of a system in light of observations, such as decision schemes for toxicity testing (Jaworska et al. 2010), species occurrence modeling (Hradsky et al. 2017), or classification of plant invasiveness based on species traits (Tyler et al. 2015).

#### Aleatory BN

An aleatory BN is a model for aleatory uncertainty in probabilistic risk assessments. An aleatory BN consists of nodes that are assessment variables, and the network itself expresses the probability of a sampling event. Hence, uncertainty about assessment parameters is outside the aleatory BN (Figure 1), and the table probabilities are the best estimates of relative frequencies for each category or interval (for discretized variables). Categorical nodes are either categorical or discrete with a finite number of states, and the table probabilities for these nodes are parameters in the risk assessment.

There are several ways to inform aleatory BNs. Assessors can specify an aleatory BN by 1) using equations and parametric probability distributions (subsequently discretized) or 2) case learning from discretized data. Borsuk et al. (2004) used an aleatory BN to link variables in an assessment. Their (aleatory) BN had been informed while considering uncertainty in parameters, as well as errors in data, using statistical models for groups of variables (nodes in the network). Carriger and Barron (2020) informed an aleatory BN using discretized samples of continuous variables from an existing probabilistic assessment model (case learning). Aleatory BNs are useful metamodels of statistical and process‐based models for groups of variables in the assessment (Varis and Kuikka 1997; Borsuk et al. 2004).

Landis et al. (2020) used mathematical equations, case learning, expert judgment, and simulation of population models to build a BN simulating the population size of Chinook salmon in different watersheds based on information about the level of a pesticide stressor and variability in dissolved O levels (ecological stressor). They derived the probability of the adverse event (population size of fewer than 500 000 individuals after 50 y) by counting the frequency with which the adverse event occurs in a sample of the node for population size.

Conditional probability distributions can be approximated using linear and nonlinear generalized regression models with fixed and random effects, which estimate parameters taking into account the relevant sources of variability and uncertainty. It is possible to implement such hierarchical models (HMs) with both frequentist and Bayesian inference (Gelman et al. 2013; Bürkner 2017). However, when expert knowledge is used, all statistical inference should be Bayesian because switching statistical principles within the same assessment may be difficult to justify (Jaynes 2003). The probability tables in aleatory BNs can be specified using equations for the HM with parametric probability distributions for estimated variability (e.g., random effects), best estimates of parameters, and without the terms representing measurement errors (see example in Borsuk et al. 2004). An alternative is to derive the table probabilities from case learning using data simulated from the HM (e.g., as in Barton et al. 2008).

**Figure 3:**
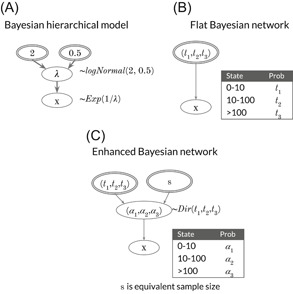
A comparison of a model of a continuous assessment variable *X*. In the Bayesian hierarchical model, variability in *X* is modeled by an exponential distribution, with the parameter *λ*. Uncertainty in the parameter is expressed by a log‐normal distribution defined by hyperparameters mean 2 and standard deviation 0.5 (**A**). An aleatory BN model expresses variability by table probabilities (*t*) on the discretized variable (states) (**B**). In the enhanced BN, uncertainty about the table probabilities (now called *α*) is expressed by a Dirichlet distribution with the hyperparameters *t* and *s* (the equivalent sample size) (**C**). BN = Bayesian network.

An enhanced BN (Figure 3) is an aleatory BN that is expanded with probability models for uncertainty about the table probabilities. These probability models are related to probability distributions for assessment parameters, which coincide only for categorical nodes or binary event nodes. The enhanced BN is a Bayesian HM (BHM) with discretized variables. For Bayesian parametric inference, the “table probability distribution” informs the priors and updates them using, for example, suitable conjugate models. We can choose to collapse the enhanced BN back into an aleatory BN by estimating the table probabilities by marginalizing over their posterior distribution. Two ways to consider uncertainty about an aleatory BN's parameters are via enhanced BNs and aleatory BNs with associated submodels (e.g., BHM), which both quantify the uncertainty about parameters (as in Borsuk et al. 2004).

#### Predictive BN

A predictive distribution is a marginal distribution for a variable considering sampling variability and the uncertainty about the sampling distribution parameters. Both frequentist and Bayesian predictive inference use predictive distributions (Geisser 1993). A predictive BN is a BN in which the probability distribution for the assessment variable nodes represents a mixture of uncertainty and variability about a future sampling event. Predictive distributions express both aleatory and epistemic uncertainty, but in an unknown combination.

A predictive BN is useful for forecasting and validating a BN against data. A predictive BN may be less useful for risk assessment because it does not explicitly reveal uncertainty about the probability of an adverse event. However, employing a predictive BN is a pragmatic approach to tackle complex assessments of future events (Pollino et al. 2007). When predictive BNs are used, we recommend communicating that there is no clear separation between epistemic and aleatory uncertainty in the assessment.

A predictive BN of assessment variables can be specified node by node, or for combinations of nodes, using statistical predictive inference outside the network or expert judgment. An alternative is to use case learning to derive the predictive BN using samples from BHMs, considering uncertainty about the parameters when sampling. A predictive BN is turned into an aleatory BN when the network is informed by data from inside the BN (case learning) (Figure 2). The BN becomes aleatory because the aposteriori probabilities are used as the best estimates of the relative frequencies of data.

Specifying a predictive distribution inside a BN requires adding parameter nodes to their associated variable, assigning parametric probability distributions for variable nodes, and specifying probability distributions for the parameter nodes. Combining uncertain parameter nodes with an aleatory BN is problematic because the parameters are continuous, because statistical predictive inference cannot be performed with a BN, and because there is a risk of updating parameter nodes when querying the network. If desired, an assessor can derive a predictive BN from an enhanced aleatory BN by marginalizing the probability distribution for the variables (instead of for the parameters) (Figure 3).

#### Bayesian models in general

Bayesian models (other than BNs) are widely used for probabilistic risk assessment; they can integrate expert judgment and data, and differentiate between aleatory and epistemic uncertainty (Kelly and Smith 2009). Bayesian inference became easier to apply with the discovery of conjugate models, in which the marginal (prior and posterior) distributions of the parameters remain in the same family when they are updated (Bernardo and Smith 1994; Gelman et al. 2013). Since the 1990s, sampling‐based approaches such as Markov Chain Monte Carlo and Approximate Bayesian Computation have allowed more complex (not necessarily conjugate) models to accommodate Bayesian updating (Fienberg 2006; Bürkner 2017; McElreath 2020). Bayesian models may become resource demanding in assessments with many linked variables and different data sources, which is a reason to use BNs instead.

## TREATMENTS OF UNCERTAINTY AT DIFFERENT LOCATIONS IN A BN APPLIED FOR RISK ASSESSMENT

Epistemic uncertainty can be associated with different locations in a risk assessment (Maxim and van der Sluijs 2011). We have identified 6 locations in the BN context where epistemic uncertainty should be treated: 1) in the structure of the network and the definition of the nodes, 2) in the parameters within the model for the assessment, 3) in the expert knowledge supporting the assessment, 4) in the data supporting the assessment, 5) in the risk management scenarios, and 6) in the output of the assessment.

Communicating epistemic uncertainty to decision makers is constrained by what and how sources of uncertainty are treated. In many cases, it is not possible to treat all possible sources of uncertainty. In that case, the assessor must acknowledge where epistemic uncertainty was not explicitly treated. For every location, it is possible to list some caveats associated with indirect epistemic uncertainty, that is, concerns about data, models, or methods supporting the assessment.

This section describes the treatment of epistemic uncertainty for BNs applied in ERA, highlighting how it is possible to separate aleatory from epistemic uncertainty. We focus on treating uncertainty associated with epistemic and aleatory BNs. We do not consider predictive BNs because they do not allow for a separation between aleatory and epistemic parameter uncertainty.

### Uncertainty located in the structure

Specifying a model's structure involves defining the nodes and the relationships between the nodes expressing causality or conditional (in)dependence within it. The development of a BN for ERA usually begins by specifying a causal model that describes the assessment question and the variables included in the assessment. Sometimes this first step, that is, the specification of the causal network, is the most valuable piece of analysis to communicate to decision makers because it may improve their understanding of the system (Fischhoff and Davis 2014).

The uncertainty associated with the structure of a BN (i.e., the network [links] and definition of nodes [including possible discretization]) is epistemic. Indirect uncertainty about the structure includes the results from the model validation of the BN and the experts' (dis)agreement about the structure and theory behind it. Further, the discretization of continuous nodes is a potential source of indirect uncertainty.

It is not possible to treat structural uncertainty within an individual BN. Instead, assessors must treat structural uncertainty by constructing alternative model structures and using separate BNs within the same assessment. One can build alternative models that differ from each other in their nodes and links (see example in Burgman 2005), or the models can also share a similar graphical form but deviate in the discretization of the nodes.

Predictions from a collection of BNs can be averaged (with probabilities over alternative structures) (Borsuk et al. 2004) or kept as a set of possible outcomes (ensemble modeling, without weights assigned to models). It is possible to apply model averaging on the output node to take into account the differences between the models. Experts can assign weights for model averaging (Fragoso et al. 2018), or if data about individual nodes are available, weights can be derived from the likelihood of each model. Alternatively, assessors can treat structurally different models as uncertainty scenarios.

### Uncertainty located in the parameters

Parameter uncertainty is relevant for BNs that include sampling variables, for which the table probabilities are fixed. For aleatory BNs, assessors must quantify the uncertainty associated with parameters external to the BN. For epistemic BNs, the network often already expresses parameter uncertainty.

Standard treatment for parameter uncertainty in risk assessment is to quantify the uncertainty using subjective probabilities or intervals and then propagate this through the output of an aleatory model using probability calculus, interval analysis, probability bounds analysis, or Monte Carlo simulation (Vose 2008; EFSA et al. 2018b). The subjective probability about relative frequency approach (Apostolakis 1990) quantifies epistemic uncertainty using subjective probabilities and uses relative frequencies for aleatory uncertainty.

For example, Borsuk et al. (2004) predicted the probability of fish kills and addressed uncertainty in this probability by quantifying uncertainty in the assessment parameters using BHMs, sampling from these uncertainty distributions, and deriving an aleatory BN for each draw of parameters. They summarized the probabilities for the outcome as a probability distribution and a probability interval. In this way, parameter uncertainty is accounted for by using multiple BNs (keeping everything the same apart from the values on the table probabilities) (Darwiche 2009). If external BHMs are not an option, assessors can treat uncertainty about parameters by embedding an aleatory BN within a model for the uncertainty of table probabilities (an enhanced BN) (Figure 3).

An alternative method to conduct an uncertainty analysis for an aleatory BN is to quantify epistemic uncertainty using intervals on the relative frequencies (table probabilities) and then searching for the lower and upper bounds of the output uncertainty. For continuous nodes, adding epistemic uncertainty as intervals would correspond to specifying a probability box (Bartell et al. 2003). For categorical nodes, one must specify intervals such that the table probabilities sum to 1. For this, one can use the imprecise Dirichlet model (Walley 1991). When uncertainty is expressed by intervals (or bounds on the probabilities interpreted as relative frequencies), the combined impact of individual parameters is evaluated using probability bounds analysis (Tucker and Ferson 2003; Burgman 2005).

A credal net (CN) is an extension of a BN representing the network's joint probability distribution by credal sets instead of a single probability distribution (Cozman 2000). Hence, a node state probability in a BN is replaced in a CN with a closed interval, which expresses the epistemic uncertainty about the probability. Although CNs have not been applied in ERA (yet), some applications in the risk assessment context can be found regarding, for instance, maritime accidents (Chen et al. 2019), fire risk assessments (Estrada‐Lugo et al. 2019), and aircraft intrusions in military areas (Antonucci et al. 2009). Several software packages exist to implement CNs, such as JavaBayes (Cozman 2000), GL2U (Antonucci et al. 2010), and OpenCOSSAN (Patelli et al. 2014).

Credal nets have also been applied to aleatory and epistemic BNs. An aleatory CN quantifies aleatory uncertainty by relative frequency and direct epistemic uncertainty by placing bounds on them. An epistemic CN adds bounds around subjective probability to consider additional weakness in the assessment, that is, indirect uncertainty. Experts elicit the bounds.

Assessors can also treat parameter uncertainty by using scenarios. For example, Landis et al. (2020) performed the risk assessment for different parameter values or model structure choices.

### Uncertainty located in expert judgment

Expert judgment is the knowledge from experts, which can inform the model structure, the assessment parameters, or the table probabilities in the BN; set uncertainty and risk management scenarios; and validate the model. Uncertainty located in expert judgment is both direct and indirect.

Experts can be biased or in disagreement. Formal expert knowledge elicitation procedures are structured to reduce common biases and mistakes experts make when providing judgments (O'Hagan et al. 2016; Hemming et al. 2018). It is crucial to specify the expert elicitation task with care because ambiguous terms can be a primary source of uncertainty in expert judgment. Thus, assessors can treat uncertainty located in expert judgment by carefully planning the expert knowledge elicitation, which includes selecting the experts.

Further, disagreement between experts is a source of uncertainty. A description of the extent to which experts disagree is a characterization of indirect uncertainty located in expert judgment. There are 2 main approaches used to aggregate expert opinion: mathematical and behavioral aggregation (O'Hagan et al. 2006). In mathematical aggregation, assessors combine the separate expert judgments by using a pooling rule, whereas experts must provide a single “consensus” judgment in behavioral aggregation.

Although a BN can express only one expert's judgment at a time, aggregation of experts may hide disagreement and relevant information about the possible answers to the assessment question. For example, when one expert judges the probability of a pest being present as 5% whereas the other judges it to be 95%, a pooled value is somewhere between these 2 probabilities. This situation is more uncertain than a situation in which both experts agree the probability is 10%.

As an alternative to aggregating judgments, one can treat (potentially disagreeing) experts' opinions as alternative models (Morgan and Mellon 2011). Expert‐specific BNs can be combined via model averaging, in which integration is based on the quantity of interest (Uusitalo et al. 2005; Lehikoinen et al. 2015) or kept separate as model ensembles or uncertainty scenarios.

### Uncertainty located in data

By data, we mean observations of a variable or a summary statistic. Uncertainty located in data includes measurement errors and weaknesses in data collection procedures. One way to treat indirect uncertainty located in the data is to conduct a quality appraisal of each data source. On the other hand, statistical HM can be used to treat direct uncertainty located in data by specifying terms for biases and measurement errors and terms for variability (e.g., random effects). Sources of variability in statistical models are important to consider because failure to do so results in uncertainty in the data or parameters.

Separate BNs, one for each data source, are required to evaluate the potential for divergent results over multiple sources of data. Then, assessors can evaluate the combined impact of uncertainty from each source by model averaging, weighting each model according to the quality rating given to each source.

### Uncertainty located in risk management scenarios

Risk management scenarios can be expressed in an assessment as hard or virtual evidence or as decision nodes. Assessors may be uncertain about the management alternatives that are to be carried out (ontological uncertainty), in what way they are to be carried out (implementation uncertainty), and how successfully they are carried out (partial controllability). Uncertainty related to risk management can have a high impact on the conclusions made from a risk assessment.

Derbyshire (2019) lists several methods to treat uncertainty in management actions, for example, specifying a time limit for when a recommended management alternative should be considered to work (to acknowledge the urgency to act in a changing system).

Uncertainty located in risk management scenarios can be treated as uncertainty scenarios by specifying alternative assessment models and searching for “robust decisions,” that is, management alternatives that are believed to work across multiple alternative futures (“robust analysis”) (Institute of Medicine 2013; Herman et al. 2015; Derbyshire 2019).

### Uncertainty located in the output

An assessment's output can be a summary of a node or a function of the nodes in the BN. Uncertainty in the output is equivalent to uncertainty about the answer to the assessment question. Both direct and indirect uncertainty should be communicated at this point.

Communicating indirect uncertainty about the output is important to help decision makers determine whether they trust the assessment or not. Indirect epistemic uncertainty can always be treated by summarizing the strength of knowledge supporting the BN. Criteria can include the strength of the evidence behind the structure of the model, the results from model calibration (e.g., goodness of fit), an evaluation of the procedure used to integrate experts' knowledge into the model, indications of expert disagreement, conflicting results from multiple studies, and the results from validation on independent data. It can also include caveats about the assumptions made during the modeling process, such as assumed causal dependencies or distributions used to describe variability. Further, the list of caveats could include the decisions made about how epistemic uncertainty should be treated, which includes documentation of the assessment model.

Direct epistemic uncertainty can be visualized and presented in tables or text. For example, Borsuk et al. (2004) communicated direct uncertainty about the probability of an adverse event (the output) using a probability density graph and a probability interval.

When communicating epistemic uncertainty in the output, one should also consider how the decision maker might respond to this uncertainty. It is useful to think of a suitable decision theory that links to the provided types and expressions of uncertainty. Bayesian decision theory (Berger 1985) applies when direct uncertainty is quantified by subjective probability, and a decision maker considers only the expected value of a decision‐relevant quantity, for example, the extinction risk. It is important to be aware that a decision maker may be sensitive to information about direct and indirect uncertainty, such as an uncertainty range for the answer to the assessment question, the results from an assessment based on uncertainty scenarios, or low confidence in an assessment. Theories about decision making in the midst of uncertainty (Herman et al. 2015) are useful to inform the decision makers about how they could respond to direct uncertainty beyond expected values.

## DISCUSSION

The terminology of uncertainty in association with applications of BNs in ERA can be confusing. Uncertainty analysis treats uncertainty from different sources, inside or outside the BN, qualitatively, or quantitatively. Both direct and indirect epistemic uncertainty located in the structure, parameters, expert judgment, data, risk management scenarios, and model output needs to be treated. There are many options to characterize uncertainties such as subjective probabilities, intervals, bounded probabilities, scenarios, or as a list of caveats. How uncertainty is treated differs between what we call epistemic and aleatory BNs. The future use of BNs for ERA should acknowledge the type of BN that is being used and that a single BN cannot treat uncertainty associated with the assessment model.

Even if both frequentist and Bayesian inference can be used to infer a BN's parameters, it is advisable to opt for Bayesian inference whenever the aim is to quantify uncertainty in the parameters by subjective probability and expert judgment is used in the assessment. The reason for this is not that BNs are called “Bayesian,” but because it is difficult to justify switching statistical principles within the same assessment (Jaynes 2003).

Uncertainty about the parameters may alternatively be expressed by bounded probabilities (resulting in 1‐ or 2‐sided intervals), which turn the BN into a CN (Cozman 2000). With an increasing supply of software packages for CNs, we expect the use of CNs in ERA to increase. It remains to be seen what the advantages of using CNs are compared to the process of embedding a BN in a probabilistic uncertainty analysis.

Our recommendation is to treat uncertainty in the structure, parameters, and data using appropriate statistical submodels before specifying the BN (as in Borsuk et al. 2004). By doing so, uncertainty analysis is performed outside the BN, and BN software is used to make queries from the aleatory BN. Besides, to encourage the use of BNs in risk assessments, efforts to extend existing software packages to support enhanced BNs and probabilistic uncertainty analysis for aleatory BNs would be beneficial.

## CONCLUSION

Failure to communicate the limitations of current knowledge in ERA may have severe consequences for decision making. The present paper develops the concept of using BNs to inform environmental risk assessment and management and contributes methodological advice for their implementation therein. We highlight potential gaps in the current treatment of uncertainty when BNs are used for risk assessments and propose a consistent framework and a set of methods for treating epistemic uncertainty. Bayesian networks are useful for ERA because they can be used to perform probabilistic reasoning based on experts' uncertainty. Also, they are a scientifically sound way to combine variables from different assessment models. Bayesian networks can model complex systems relatively quickly by discretizing variables and using best estimates of assessment parameters.

However, it is critical to be aware of the limitations of treating epistemic uncertainty in a single BN. A systematic approach to treat uncertainty when applying BNs in ERA should 1) describe the nature of the BN used for ERA, 2) differentiate between aleatory and epistemic uncertainty, 3) acknowledge uncertainty at different locations in a risk assessment, 4) use appropriate statistical models and expert elicitation to quantify uncertainty, and 5) communicate direct and indirect uncertainty.

We hope our recommendations serve to address one of the greatest challenges in environmental risk assessment: to acknowledge things we do not know and be confident about our uncertainty (Spiegelhalter and Riesch 2011).

## SUPPLEMENTAL DATA

Supplemental Data show the terminology for uncertainty and BNs in risk assessment used in this paper.

## Supporting information

This article contains online‐only Supplemental Data.

Supporting information.

Supporting information.

## Data Availability

There are no data or specific tools used in this paper. Contact corresponding author Ullrika Sahlin (Ullrika.Sahlin@cec.lu.se) for requests for data.
