# Supplementary material for: “This Is What We Don't Know”: Treating Epistemic Uncertainty in Bayesian Networks for Risk Assessment
Source: Integr Environ Assess Manag. 2020 Dec 3;17(1):221–32. doi: 10.1002/ieam.4367 (PMC7839433; doi:10.1002/ieam.4367)
Supplement: Supplementary file 1 — Supporting information. [file IEAM-17-221-s001.docx]

**Supporting Information**

Title: ‘This is what we don't know’ – Treating epistemic uncertainty in Bayesian networks for risk assessment

Authors:

**TERMINOLOGY**

There exist some overlaps in the terminology used in the BN literature compared to ERA literature. For the purpose of this paper we adopt the following terminology:

**Assessment**

“Assessment question” is the statement, hypothesis or decision which the assessment is made to elucidate.

“Assessment variables” are quantities that take values according to a random process.

“Assessment parameters” are constructs within a specification of a scientific model that have true values (given the model) about which we are typically uncertain. They are typically unobservable.

“Assessment output” is the derived quantity that answers the assessment question.

“Assessment model” is a specification of the assessment variables, with assessment parameters as inputs, that predict or estimate quantities that are needed to answer the assessment question.

“Data” is an observation of a variable.

“Expert judgement” is information from experts about their subjective belief on uncertainty in a quantity, typically elicited by a structured process.

**Uncertainty**

“Uncertainty analysis” is the process of identifying limitations in knowledge and evaluating their implications for conclusions.

“Epistemic uncertainty” is a limitation in knowledge.

“Direct epistemic uncertainty” is limitations in knowledge about the characterisation of the facts, numbers or scientific hypotheses.

“Indirect epistemic uncertainty” is the strength in our knowledge about the facts, numbers and scientific hypotheses.

“Aleatory uncertainty” (a.k.a. variability) is inherent randomness in a quantity.

“Structural uncertainty” is epistemic uncertainty located in the structure of the assessment model. It is often also called model uncertainty.

“Parameter uncertainty” is epistemic uncertainty located in the assessment parameters.

“Uncertainty location” is a part of an assessment containing uncertainty

**Bayesian network**

A “Bayesian network” (BN) is a probability distribution over nodes linked by edges, expressing conditional dependencies.

“Table probabilities” are the probabilities inside the nodes’ probability tables (PTs) and conditional probability tables (CPTs).

“BN node” is a BN representation of an assessment variable.

“Categorical node” is a BN node representing a finite number of distinct, not necessarily ordered, categories.

“Continuous node” is a BN node representing a variable specified by a continuous, most often parametric, probability distribution. Continuous nodes can be statically or dynamically discretized.

“Probabilistic reasoning” is Bayesian inference over nodes given an instantiation of a BN.

A “node state probability distribution” is the marginalised probability distribution of a node, which may change when a network is instantiated with BN evidence.

“Evidence”, in the context of a BN, is a categorical probability distribution imposed onto a BN node to override node state probabilities. It is most commonly referred to as “virtual evidence”, unless one of the categories is assigned probability of one (1); then it is called “hard evidence”.

“Instantiation” (a.k.a. a case or finding) is a combination of evidence on one or several nodes of a BN.

“BN prediction” is the node state probability distribution(s) in a network instantiated by evidence.

Epistemic BN is a BN consisting of nodes for quantities we are uncertain about and nodes for data that are children to the other nodes. Epistemic BNs are used for probabilistic reasoning of the state of uncertain quantities in light of evidence.

Aleatory BN is a BN of a sampling event, where all nodes are variables and the probability distribution express variability in these nodes.

“Enhanced BN” is a BN where probability distributions are added to aleatory BN to express epistemic uncertainty in the table probabilities.

Predictive BN is a BN for a future sampling event, where nodes are variables and the probability distribution is a mixture of epistemic and aleatory uncertainty.

Bayesian Hierarchical Model is a joint distribution over variables and parameters structured based on conditional dependencies given a direct acyclic graph of the model.
