# Supplementary material for: “This Is What We Don't Know”: Treating Epistemic Uncertainty in Bayesian Networks for Risk Assessment
Source: Integr Environ Assess Manag. 2020 Dec 3;17(1):221–32. doi: 10.1002/ieam.4367 (PMC7839433; doi:10.1002/ieam.4367)
Supplement: Supplementary file 2 — Supporting information. [file IEAM-17-221-s002.docx]

# SUPPLEMENTARY INFORMATION

The terminology for uncertainty and BNs in risk assessment used in this paper.
